# Education and Disease

**Published:** 1895-01-19

**Authors:** 


### Education and Disease.

The problem how to educate the children without killing them is not so simple as it might seem. The interesting address lately delivered by Dr. Shirley Murphy before the Epidemiological Society seems to clench what has been often said before—that school life is responsible for a great deal of the infectious disease from which children suffer. The relation of diphtheria to elementary education is one which it is impossible to ignore. Taking the London figures as given by Dr. Murphy, it appears not only that the incidence of diphtheria is greatest in children at school age, *i.e*, between three years old and ten, but that when the general diphtheria mortality varies, that of the school children lags behind when there is improvement and takes the lead when things are getting worse. Thus between 1871-80 the general mortality was less than in the preceding ten years, but this fall in mortality did not equally benefit all the age groups. For some reason, children from three to ten years of age shared less in this improvement than those under three, or persons above ten, suggesting that about that time some new factor had come into existence which tended to maintain the diphtheria prevalence among children of school age.

But in the next ten years the mortality at all ages had increased. Not equally, however, for the increase among children from three to ten was greater than that among those either below or above that time of life.

Again, taking the years 1891 to 1893, during which diphtheria has been specially prevalent, and comparing them with periods before 1870, we again find that the increase was chiefly due to a greater prevalence among the children, and especially among those of school age. From all this the conclusion is drawn that the Elementary Education Act, which first became operative in 1871, has been an important agent in promoting the dissemination of infection. The point of interest, however, in Dr. Murphy's address lay in the further demonstration of school influence, as shown by the effect of the holidays on the prevalence of disease. The summer holidays take place at a time when the curves both of scarlet fever and diphtheria are on the rise, and if infection is caught at school there ought at holiday times to be some break in the curve for children of school age, a diminution of its steepness or an actual fall. This proves to have been the case in regard to scarlet fever both in 1892 and 1893. Again, in regard to diphtheria, the same relations has been shown to exist, the holiday depression being well seen in 1893. Dr. Murphy also noticed that the holiday depression in the scarlet fever curve occurred a week later in 1893 than in 1892, in that point corresponding to the fact that the holidays themselves were a week later in 1893. We cannot, then, avoid the conclusion that school attendance is a factor, and a not inconsiderable one, in the dissemination of infectious diseases, and the problem how to lessen this danger of school life is one worthy of the most careful attention of all interested in education. The rough, crude cry of the "man in the street" is "close the schools." That, however, is often only a postponement of the difficulty, and is a measure which is looked on with increasing dislike both by educationalists and sanitarians. We must first inquire how, and by what mechanism, schools become foci of infection, and here we cannot shut our eyes to the experience of some hospitals in regard to the infection of diphtheria. It was recently stated by Dr. Washbourne and in fact it is known to be the case, that when ventilation is free, the beds not too close together, and the discipline good, the disease but rarely attacks non-diphtherial cases accidentally sent to diphtheria wards.

The difficulties in schools are manifold, and it is not easy to see a way of escape from them. For teaching purposes, especially with the large classes now in vogue, the children must be crowded together so as to be near the teacher and the black-board, and however ample the cubic space and free the ventilation of the room, neither space nor ventilation is sufficient in that portion of the room occupied by the crowd of children, nor is it easy to see how it can be made sufficient.

The managers and architects of schools are not, however, solely responsible for the dangers of school life. The habits of the children are often conducive in the highest degree to the conveyance of infection, and especially of such an infection as that of diphtheria. The use of saliva in washing slates, where slates are used, the sucking of pencils, the community of lollypops, the exchange of hankerchiefs, the kissing and fondling of each other so commonly seen among the infants are all channels of infection obvious enough but hard to stop. The freest ventilation, the largest cubic space, and the most perfect discipline must be provided, but with all that we fear that the only feasable method of diminishing school infection will be by a much more perfect method of inspection of the children and their throats than can be expected from the teachers themselves. A carefully arranged medical inspection, coupled with the provision of isolation rooms in which doubtful cases might be placed pending the arrival of a doctor, wonld do much to lessen one of the chief dangers of school life ; but to be of real use it must be done in a thorough manner, and that means money.

